# Super-strong materials for temperatures exceeding 2000 °C

**DOI:** 10.1038/srep40730

**Published:** 2017-01-19

**Authors:** Laura Silvestroni, Hans-Joachim Kleebe, William G. Fahrenholtz, Jeremy Watts

**Affiliations:** 1 CNR-ISTEC, Institute of Science and Technology for Ceramics, Via Granarolo 64, I-48018 Faenza, Italy; 2 TUD-IAG, Institute of Applied Geosciences, Schnittspahnstraße 9, D-64287 Darmstadt, Germany; 3 Department of Materials Science and Engineering, Missouri University of Science and Technology, Rolla, MO 65409, USA.

## Abstract

Ceramics based on group IV-V transition metal borides and carbides possess melting points above 3000 °C, are ablation resistant and are, therefore, candidates for the design of components of next generation space vehicles, rocket nozzle inserts, and nose cones or leading edges for hypersonic aerospace vehicles. As such, they will have to bear high thermo-mechanical loads, which makes strength at high temperature of great importance. While testing of these materials above 2000 °C is necessary to prove their capabilities at anticipated operating temperatures, literature reports are quite limited. Reported strength values for zirconium diboride (ZrB_2_) ceramics can exceed 1 GPa at room temperature, but these values rapidly decrease, with all previously reported strengths being less than 340 MPa at 1500 °C or above. Here, we show how the strength of ZrB_2_ ceramics can be increased to more than 800 MPa at temperatures in the range of 1500–2100 °C. These exceptional strengths are due to a core-shell microstructure, which leads to *in-situ* toughening and sub-grain refinement at elevated temperatures. Our findings promise to open a new avenue to designing materials that are super-strong at ultra-high temperatures.

The quest for novel materials with improved structural capabilities is always a central challenge to materials science. In particular, high melting point compounds are required in the aerospace and propulsion fields^1^,^2^. ZrB_2_ has been widely considered a potential candidate in the ultra-high temperature regime, i.e. above 1500 °C^2^,^3^, thanks to its unusual combination of physiochemical properties. However, the utilization of pure borides is limited by three major factors: i) the strong covalent bonds hinder its densification, which has previously precluded realization of the true potential of ZrB_2_ as a structural material; ii) its oxidation resistance, since ZrB_2_ oxidation generates ZrO_2_, which is porous and non-protective, and B_2_O_3_, which readily evaporates; and lastly iii) it is brittle and susceptible to thermal shock failure. The addition of secondary phases is essential to mitigate these shortcomings. During the design of ZrB_2_-based ceramic composites, two main aspects have to be considered: the distribution of the secondary phase, continuous or discontinuous, and the melting point of the second phase or eutectic temperature between ZrB_2_ and the second phase that limit the maximum temperature at which these can be used. In this respect, a large variety of ZrB_2_ ceramics containing silicon carbide (SiC) or transition metal silicides have been generated with the aim of bridging the above mentioned gaps. Using these additives, improvements have been demonstrated^3^,^4^,^5^,^6^.

Very limited data are available on the mechanical behavior above 1500 °C, as few facilities are equipped to perform such testing. A selected collection of recent data concerning ZrB_2_ composites tested up to ultra-high temperature is reported in Fig. 1. These studies show that the strength of ceramics containing 20–30 vol% SiC is generally retained, or even increased, from room temperature up to 1000 °C. Strengths range between 500 and 700 MPa due to the formation of a continuous silica-based-glass capable of healing surface flaws^7^,^8^,^9^,^10^,^11^. However, by 1500 °C, strength decreases to 200–340 MPa, owing to coalescence of micro-voids formed at triple junctions, softening of the grain boundary phases, and grain coarsening^4^,^5^,^6^.

Tungsten additions to ZrB_2_ and HfB_2_ based ceramics have been examined by several research groups, in view of substantial enhancements in oxidation resistance and bending strength up to 1600 °C^12^,^13^,^14^,^15^,^16^,^17^. Figure 1 shows that the addition of 5 vol% of WC to a ZrB_2_–20 vol% SiC composite resulted in strengths above 600 MPa at 1600 °C when measured in argon^12^. Although several hypotheses have been formulated, the role of W additions has not yet been fully explained.

In this paper, we report the flexural strength at temperatures up to 2100 °C for a ZrB_2_ ceramic containing WC and discontinuous SiC particles. The strength values were exceptional, having never before been achieved at such temperatures, as shown in Fig. 1 (red line). We suggest that W-doping plays a fundamental role in the strength retention, as it promotes a unique combination of favorable conditions both in the outermost surface and in the bulk. The outer surface of the ceramic is toughened by finely dispersed metal W particles in the oxide scale. In the bulk, the microstructure is characterized by a core-shell morphology and refractory phases at triple junctions, which result in grain refinement due to the formation of sub-grain boundaries via dislocation networks.

## Microstructure of the as-sintered ceramic

Fully dense ZrB_2_ containing 5 vol% WC and 3 vol% β-SiC (henceforth denoted ZS3-WC) was prepared by hot pressing. The diboride matrix was uniform with a mean grain size of 1.8 ± 0.6 μm, Fig. 2. The matrix developed a sub-structure, widely known as “core-shell”, in which grains were comprised of a stoichiometric ZrB_2_ core surrounded by an isostructural (Zr,W)B_2_ solid solution shell. The amount of W in the shell was in the range of 2–4 at% and the shell comprised ~30% of the volume of the ZrB_2_-based matrix, based on estimation using image analysis of polished surfaces. Our ZS3-WC ceramic also contained minor phases including the added SiC particles along with WB, a mixed (Zr,W)C solution, and C that were formed during hot pressing by reactions with the starting ZrB_2_ powders and/or the native oxides covering them^17^.

## Mechanical properties

**Figure 1:**
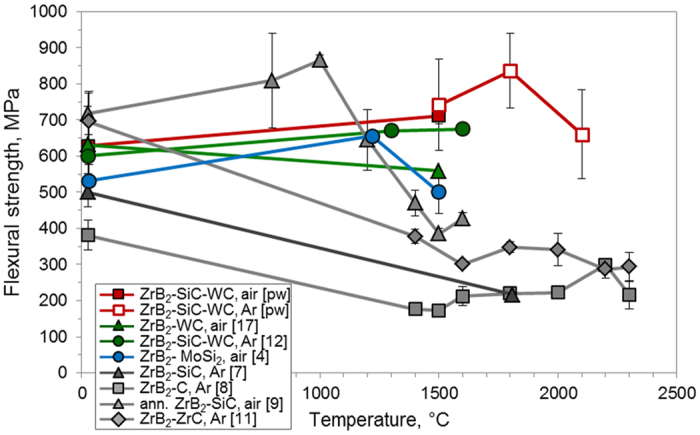
Flexural strength as a function of temperature for the highest strength ZrB_2_-ceramics^4^,^12^,^17^, or tested at the highest temperatures in air or Argon^7^,^8^,^9^,^11^. pw: present work.

**Figure 2:**
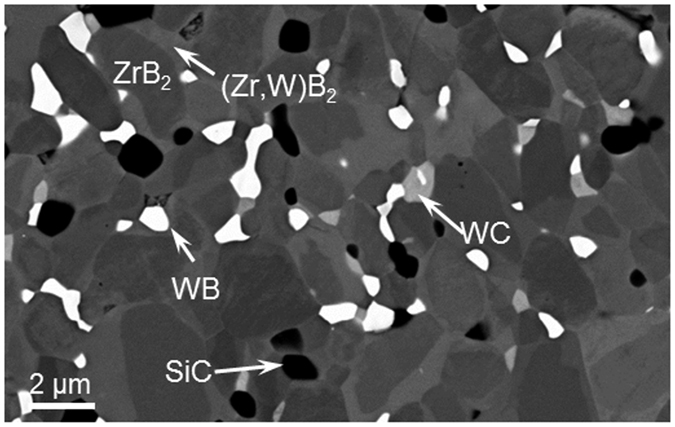
SEM image showing a representative view of the as-sintered microstructure of ZS3-WC with the core-rim structure of the boride matrix and the main secondary phases.

The strength of ZS3-WC as a function of temperature is plotted in Fig. 1 and, compared to previously published data on other ZrB_2_-based ceramics^4^,^7^,^8^,^9^,^11^,^12^,^17^. Our ceramic possesses the highest strength ever reported for a ZrB_2_-based ceramic at temperatures above 1500 °C. The room temperature strength of ZS3-WC was 630 ± 76 MPa, similar to other fully dense ZrB_2_ or HfB_2_ ceramics containing W-compounds or other refractory sintering additives^3^,^7^,^8^,^9^,^10^,^11^. Increasing the testing temperature to 1500 °C, the strength was ~700 MPa in either air or argon. Remarkably, the ceramic had average strengths of 836 ± 103 MPa at 1800 °C and 660 ± 123 MPa at 2100 °C, which are approximately double the highest previously reported values for ZrB_2_-based ceramics at those temperatures^7^,^8^,^9^,^10^,^11^,^12^.

Fracture toughness measured by chevron notched beam method at room temperature was 3.7 ± 0.9 MPa · √m. When we tested this property at 1500 °C we did not observe a substantial increase: 3.455 ± 0.4 MPa · √m in air and 4.6 ± 0.5 MPa·√m, when measured in Argon atmosphere.

## Fractographic analysis

**Figure 3:**
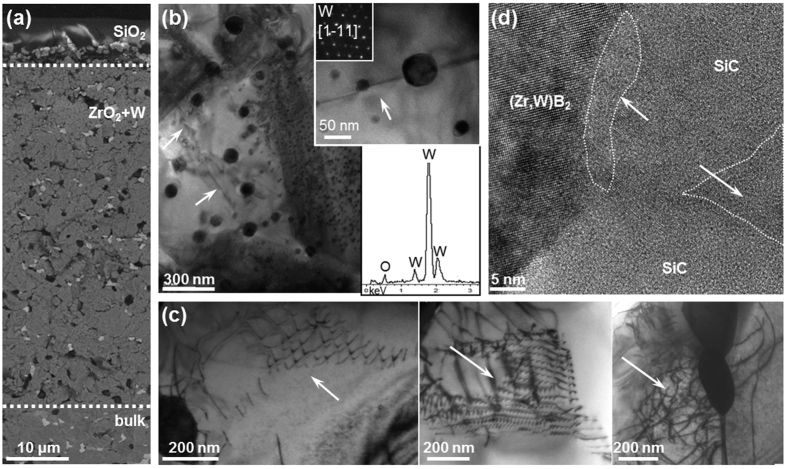
Fractographic analysis of ZS3-WC after bending test at 1500 °C in air showing (**a**) an SEM image of the cross section of the modified surface scale, (**b**) TEM images showing the W nanoparticles encased in ZrO_2_ grains with diffraction pattern and an EDS spectrum showing the cubic structure and composition of the inclusions (arrays indicate dislocations front pinned by the nano-sized W), (**c**) examples of the boride grains in the bulk with sub-grain boundaries consisting of hexagonal dislocation networks indicated by arrows, (**d**) HRTEM image pointing to amorphous phase close to a SiC/SiC interface.

*Air, 1500* °*C* – After testing in air at 1500 °C, Fig. 3a, an oxide scale ~60 μm thick formed. The outermost layer was glassy SiO_2_ about 7 μm thick. The next layer consisted of interconnected ZrO_2_ grains, and was ~55 μm thick. This layer showed intergranular fracture, in contrast to transgranular failure observed in the bulk. Within this scale, some partially oxidized SiC grains with dark contrast and W-based particles with bright contrast were present. The WB grains that were at the triple junctions of the hot pressed ceramic remained unaltered within this oxide scale, because they have higher oxidation resistance than ZrB_2_^17^,^18^.

Looking closer in the oxide scale, we found roughly spherical W inclusions in ZrO_2_ grains through the whole layer Fig. 3b (for more details on the thermodynamics of the Zr-W-O system, see the Supplementary Information). Similar to a previous study of the oxidation of a ZrB_2_-WSi_2_ ceramic at 1650 °C^18^, we observed that the (Zr,W)B_2_ solid solution oxidized to form ZrO_2_ that encased these dispersed W nanoparticles, which ranged from 5 to 60 nm in diameter. High resolution images, coupled with chemical and structural analyses, revealed ZrO_2_ to be monoclinic and chemically pure, i.e. without any trace of W in solution, which is consistent with the negligible solubility of W in zirconia^19^. Most importantly, we found that the W metal nanoparticles acted as pins for the advancement of dislocation fronts inside ZrO_2_, which is highlighted by arrows in the inset of Fig. 3b.

Analysis in the bulk of the broken bars showed that ZrB_2_ grain size remained about the same, at around 2.2 ± 0.8 μm. Residual carbon sheets and WB were observed to pull-out of the surface during fracture. We performed further microstructural analysis on this specimen by TEM to disclose modifications in the bulk at the nano-scale. The matrix grains in the bulk displayed hexagonal dislocation networks and generally we observed high dislocation density across the grains, like the example in Fig. 3c. TEM images of the secondary phase arrangement in the bulk confirmed that ZrB_2_, WB and SiC were tightly bonded to each other and in particular, SiC and WB displayed systematic stacking faults contrast. Investigation of the grain boundaries in high resolution mode revealed that, even at this temperature, interfaces between adjacent (Zr,W)B_2_ shells and (Zr, W)B_2_/WB and WB/SiC grain interfaces were free of amorphous phases. In contrast, we found traces of a glassy phase at SiC/SiC interfaces, Fig. 3d, indicating that oxidation and grain sliding could be triggered in SiC-rich areas.

**Figure 4:**
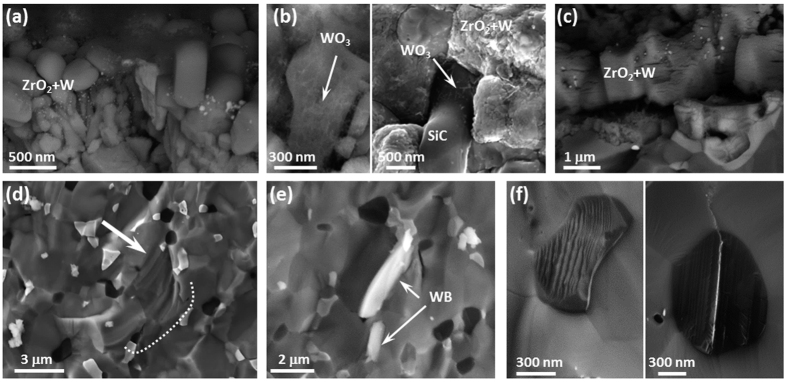
Fractographic analysis after bending tests in argon at 1500–2100 °C showing (**a**) the typical outermost oxidized scale upon tests at 1500 and 1800 °C, (**b**) WO_3_ whiskers surrounding ZrO_2_ and SiC grains, (**c**) extended cracking in the oxidized scale after testing at 2100 °C, (**d**) elongation and deformation of boride grains in the matrix after testing at 2100 °C indicated by the arrow, (**e**) WB pullout from the bulk and (**f**) SiC particle surfaces after crack propagation perpendicular and parallel to the stacking faults, left and right respectively.

*Argon, 1500*–*2100* °*C* - Fractographic analysis of specimens tested in argon at temperatures of 1500 °C to 2100 °C had no SiO_2_ layer on the fracture surface. Only ~5 μm of the outer surface was oxidized, presumably due to trace impurities of oxygen in the test environment, Fig. 4a.

The morphology and thickness of the external layer did not vary notably with increasing temperature, confirming comparable levels of residual oxygen in the testing chamber, which has previously been estimated to be in the range of 10^−12^ atm to 10^−14^ atm^20^. Interestingly, ZrO_2_ grains were adorned with W nanoparticles. In addition, WO_3_ whiskers surrounded both ZrO_2_ and SiC grains, Fig. 4b. The oxide scale started to display cracks upon cooling when the test temperature was 2100 °C, Fig. 4c, probably due to the large coefficient of thermal mismatch between the boride matrix (~7 × 10^−6^/K^21^) and the oxide zirconia (10.3 × 10^−6^/K^22^) and to the tetragonal to monoclinic transformation of zirconia.

In the bulk, ZrB_2_ grain size remained about the same as the as-sintered ceramic, ~2.2 μm. The grains also maintained a rounded shape until ~1800 °C. Above this temperature, the microstructure of the unoxidized bulk changed. The aspect ratio of the boride grains increased resulting in an elongated shape perpendicular to the loading direction, as indicated by the arrow in Fig. 4d, suggesting plasticity at the test temperature. At all elevated test temperatures, WB grains frequently pulled-out from the matrix, as shown in Fig. 4e. We also noticed that when SiC grains were fractured perpendicular to stacking faults, the grain surface was rough and stepped, indicating crack deflection at the nanostructural level, Fig. 4f left, whilst crack propagation parallel to the stacking faults proceeded straight without deflection, Fig. 4f right.

## Discussion

Ceramics tested in either air or argon had a combination of favorable features that prevented the decrease in strength that typically has been observed as temperature increased. The following discussion describes the different mechanisms that we believe are responsible for the experimentally observed increase in strength at elevated temperatures.

The elevated temperature failure of fine-grained polycrystalline ceramics generally occurs by nucleation, propagation, blunting and coalescence of cracks^23^. Cracks in ceramics nucleate preferentially at microstructural and/or chemical inhomogeneities, where the local stress concentrations occur. In this respect, the roughness of the tensile surface of the test specimens is of paramount importance, as flaws induced by surface finishing act as macroscopic discontinuities. Other inhomogeneities that affect strength can include grain boundaries, precipitates, or second phases. For example, grain growth at elevated temperatures or preferential oxidation of second phases can reduce strength by increasing flaw size. Likewise, amorphous phases that are continuous or segregated at the grain boundaries can lead to viscous flow or solution/reprecipitation mechanisms that further reduce strength^24^.

Strength (*σ*) retention at high temperature has previously been attributed to various phenomena such as: (i) release of internal residual stresses^25^; (ii) formation of a protective oxide scale^26^; or to changes in one of the parameters of Griffith’s law (Equation 1) like (iii) an increase in fracture toughness (*K*_*Ic*_); or (iv) a decrease in flaw size (*a*) by grain refinement:


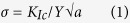


where *Y* is a shape factor constant.

- Relaxation of thermal residual stresses is common for all of the testing temperatures in this study. Previous research on ZrB_2_-SiC contaminated with WC showed that residual stresses only begin to accumulate as temperature decreases below 1400 °C^27^. As a result, residual stresses should be negligible at all testing temperatures in the present study and will be ignored in this discussion.
- Some previous studies have attributed an increase in strength at moderate temperatures to the formation of a continuous oxide scale that can heal surface flaws^3^,^4^,^5^,^6^. While testing in air at 1500 °C leads to the formation of an outer protective layer, Fig. 3a, we dismiss this mechanism for the further increase in strength at higher temperatures since a continuous SiO_2_ layer did not form when we tested the ceramic in argon and its strength also increased.
- A phenomenon common to all of the materials tested in the present study was the formation of a densely packed region of ZrO_2_ grains containing finely distributed tungsten nanoparticles, Fig. 3b. The homogeneously dispersed W nanoparticles could promote ductile-phase toughening of the ZrO_2_ layer, due to ductile-ligament bridging in the crack wake, or crack bowing^25^. Tungsten exhibits a ductile-brittle transition temperature (DBTT) below which little or no macroscopic plastic deformation occurs prior to fracture^28^. In contrast, above the DBTT, the fracture toughness of metallic W increases abruptly, up to as high as ~100 MPa · √m^29^. Hence, above the DBTT, ductile W inclusions can bridge the crack and stretch as the crack opens, thus absorbing energy and increasing fracture toughness^30^. Nawa *et al*.^31^ suggested that a decrease in flaw size, associated with the interpenetrated nanostructure, combined with a stress shielding effect, created near the crack tip by the elongated metal crystals bridging the crack, were the main mechanisms responsible for an increase in strength and fracture toughness for a ZrO_2_ ceramic containing nano-dispersed Mo particles. Similarly, Sekino *et al*. found that 5–10 vol% of finely dispersed W nanoparticles enhanced the fracture strength of Al_2_O_3_ by decreasing grain size and producing highly localized residual stresses due to the thermal expansion mismatch between matrix and inclusions^32^. While the presence of W nanoparticles in ZrO_2_ grains is a plausible mechanism that could lead to an increase in both strength and toughness with increasing temperature, all specimens tested in argon displayed similar or even higher strengths than those tested in air at 1500 °C despite having a notably thinner scale, about 5 μm after testing in argon, Fig. 4a, compared to about 55 μm after testing in air, Fig. 3a. In both cases, the toughened material constitutes a minimal fraction of the overall test bar. However, the toughened material is located on the outer surface, i.e. where cracks initiate. In addition, as little as 5 vol% of a toughening phase (fiber, whiskers, secondary phases) can improve the toughness of brittle ceramics by as much as 30–40%^33^. Because of the materials tested in argon had a thinner toughened ZrO_2_ layer and higher strengths at increasing temperatures, we believe that the toughened ZrO_2_ layer plays only a minor role in the increased strength with increasing temperature. An increase in the fracture toughness of the bulk ceramic could also lead to an increase in strength with increasing temperature. The microstructure of the bulk was not altered significantly by increasing test temperature, but pullout of WB particles or C pockets, which indicate weake grain boundaries, could provide additional toughening as temperature increases. Likewise, a peculiar fracture mode was observed for SiC particles, which appears to be associated with stacking faults. Partial dislocations and stacking faults have been reported to act as preferred crack nucleation sites and propagation paths during deformation, since the bonding across the faulting plane is weakened due to the irregular rearrangement of bonds between boundary atoms^34^. Hence, cracks could dissipate more energy as they change direction when interacting with SiC particles. Similar stacking fault features were also observed in WB regions. Together, SiC and WB make up ~8 vol% of the ceramic where cracks could potentially dissipate more energy, if they meet inclusions in the proper orientation. However, since these mechanisms should be equal in all of the ceramics regardless of the testing atmosphere, this mechanism does not explain the increased strength for ceramics tested in argon. Hence, this mechanism may be active, but it is thought not to be the main reason for increased strength with increasing temperature. As confirmation, the fracture toughness measured at room temperature was not statistically different to that measured at 1500 °C in air or Argon.
- Grain refinement associated with the core-shell microstructure is the most plausible mechanism for the remarkable strength at elevated temperature for the ZS3-WC ceramics. The W-rich shells make up more than 30 vol% of the composite. The refractory shells appeared to pin grain growth and effectively prevent creep at elevated temperatures. In addition, all the grain boundaries were free of amorphous phases except adjacent SiC particles, which rarely occurred due to the low volume fraction of SiC and its good dispersion in the matrix.

The as-sintered matrix is characterized by (Zr,W)B_2_ solid solution shells surrounding ZrB_2_ cores. During testing at elevated temperatures, dislocations accumulated at the interfaces between cores and shells. The core/shell morphology is particularly suited to absorb energy during deformation according to the “core-mantle” model proposed by Gifkins^35^, which hypothesizes that dislocations movement only occurs in the mantle region of the grain, i.e. in the region close to the grain boundaries. In ZS3-WC, this would be the (Zr,W)B_2_ solid solution shells. This behavior can benefit elevated temperature strength, as it enables plastic deformation through dislocation movement and accommodation of mechanical stress during loading. By this model, secondary dislocations would interact with the pre-existing dislocations forming grids, complex tangles with high dislocation density or polygonised dislocation walls. Each of these regions could then, in turn, act as a new obstacle to further slip. Since each slip line is stopped at another, the dislocations should tend to cluster into local regions forming cell structures, thus inducing sub-grain refinement as shown in Fig. 3c. Similar behavior, leading to a Hall Petch hardening^36^ in the rims, had been observed also in TiCN cermets^37^. However, at high temperature, generally intragranular deformation occurs in the whole grain and dislocation pile-up takes place at the rim-rim interface resulting in grains de-cohesion, which is one reason why strength increases are not typically reported at elevated temperatures for these ceramics.

The highest average strength of 836 ± 103 MPa (maximum of 940 MPa for one specimen) was achieved at 1800 °C. Above this temperature, the strength decreased to ~660 MPa at 2100 °C, which is still in the same range of the room temperature values, 630 MPa, Fig. 1. By 2100 °C, some critical temperatures for the phases present in the system had been passed resulting in several changes that could be responsible for the loss of strength including:

- α-SiC tends to grow along the *c*-direction and form platelets, which could increase the flaw size;
- the eutectic temperature between ZrO_2_ and Zr is 2065 °C^38^, which could lead to liquid formation and weakening of the structure;
- other eutectic temperatures are approached or passed (between 2050 and 2100 °C for ZrB_2_ and WB, 2230 °C for ZrB_2_ and C, or at ~2270 °C for WB and C^39^) which could lead to grain sliding.

In summary, ZS3-WC is a remarkable example of ceramic material that maintains its strength up to 2100 °C. We attribute this behavior to a combination of mechanisms, the most important of which are associated with the core-shell microstructure (grain refinement owing to hexagonal dislocation networks), but also include toughening contributions from metal nanoparticles and pullout of WB and C inclusions. Average strengths as high as ~830 MPa were observed at 1800 °C, which make this material highly attractive for applications at ultra-high temperatures. Further, identification of the role of the core-shell structure provides a potential design criterion for a new paradigm of ultra-high temperature structural materials.

## Methods

### Ceramic Preparation

A ZrB_2_ ceramic composite containing 5 vol% of WC and 3 vol% of β-SiC (ZS3-WC) was produced using the following commercial powders: ZrB_2_ (H.C. Starck, grade B, Germany), purity >98 wt%, mean particle size: 2.1 μm, impurities (wt%): C 0.25, O 2, N 0.25, Fe 0.1, Hf 0.2; WC (Treibacher Industries AG, grade SD0.5, Germany), purity 99.5 wt%, mean particle size: 0.5 μm; and SiC (H.C. Starck, grade BF-12, Germany), purity >99 wt%, impurities (wt%): O 0.9.

The base component (ZrB_2_) and secondary phases (SiC, WC) were batched into a PET bottle and then ball-milled for 24 hours in absolute ethanol using SiC milling media. Subsequently the slurries, dried using a rotary evaporator, were sieved through a 150 μm metallic screen. A 30 mm diameter pellet was cold compacted using a uniaxial press with 20 MPa applied pressure. The *green* pellet was directly positioned into the graphite die located inside the operating vacuum chamber of the hot-press. The inner walls of the graphite die were protected by a BN-sprayed graphitized 0.5 mm thick foil. The pellet was then hot-pressed in vacuum environment (~0.1 mbar) using an induction-heated graphite die and applying a constant uniaxial pressure of 30 MPa while ramping up to 1930 °C using a 15 °C/min heating rate. Once the target temperature was reached, the uniaxial pressure was increased to 40 MPa and held up to 40 minutes to promote full densification. The temperature was monitored by means of an optical pyrometer focused into a blind hole drilled on the external surface of the die. At the end of the dwell, the furnace power was turned off and the specimen was allowed to cool naturally.

### Microstructure characterization

The bulk density was measured by Archimedes’ method and confirmed by examination of polished cross section. The microstructures of the as-sintered ZrB_2_ ceramic and specimens after high temperature bend testing were analyzed on fractured and polished surfaces by field-emission scanning electron microscopy (FESEM, mod. ΣIGMA, ZEISS NTS Gmbh, Germany) coupled to an energy dispersive X-ray micro-analyzer (EDS, mod. INCA Energy 300, Oxford instruments, UK).

Specimens for transmission electron microscopy (TEM) were prepared by cutting 3 mm discs from the tested bars. The specimens were mechanically ground to a thickness of about 20 μm and then further thinned by ion beam milling until perforations were observed by optical microscopy. Local phase analysis was performed using TEM (JEOL JEM 2100 F) operating at a nominal voltage of 200 keV and equipped with an energy-dispersive X-ray system (EDS, mod. INCA Energy 300, Oxford instruments, UK). Electron diffraction pattern identification was carried out through the software tool developed for Digital Micrograph^40^.

Key microstructural features like residual porosity, mean grain size and volumetric content of the secondary phases were evaluated using FESEM micrographs and a commercial image analysis software package (Image Pro Plus, v.7, Media Cybernetics, USA).

### Mechanical characterization

The 4-pt flexure strength (*σ*) was measured at room temperature (RT) and at 1500 °C in air, using the guidelines of the European standards for advanced ceramics ENV843-1:2006 and EN820-1:2002, or in the 1500–2100 °C temperature range in protective Ar atmosphere, following the ASTM standard C1211. Chamfered type-A bars with dimensions 25.0 mm × 2.5 mm × 2.0 mm (length by width by thickness, respectively) were tested at RT using a semi-articulated steel 4-pt fixture (lower span 20mm, upper span 10 mm) in a screw-driven load frame (Zwick-Roell mod. Z050, Germany), 1 mm/min of cross-head speed. Flexure strength at 1500 °C in air was instead measured using an Instron apparatus (mod. 6025) equipped with a 4-pt fixture made of SiC, whilst high temperature strength in Ar was measured using a fully articulated graphite fixture in a mechanical testing apparatus consisting of a screw-driven instrumented load frame (33R4204, Instron, Norwood, MA), induction heated (SI-30KWLF, Superior Induction Technology, Pasadena, CA) graphite hot zone inside an environmental chamber. When specimens were tested in protective environment, the chamber was purged with argon for an approximately 90 min prior to heating. Before applying the load during testing at high temperature, a dwell of 5 min was set to reach thermal equilibrium. For each testing temperature, at least 3 bars were tested. The crosshead rate was 1 mm/min at room temperature and at 1500 °C, 0.2 mm/min at 1800 °C and 0.5 mm/min at 2100 °C.

Fracture toughness (K_Ic_) was evaluated using chevron notched beams in flexure. The test bars, 25.0 mm × 2.0 mm × 2.5 mm (length by width by thickness, respectively) were notched with a 0.1 mm thick diamond saw; the chevron-notch tip depth and average side length were ~0.12 and 0.80 of the bar thickness, respectively. The specimens were fractured using a semi-articulated alumina four-point fixture with a lower span of 20 mm and an upper span of 10 mm using the Instron screw-driven load frame mentioned above. The high-temperature tests were carried out in argon protective gas. Before the bending test, a soaking time of 18 min was set to reach thermal equilibrium. The specimens, three for each composite, were loaded with a crosshead speed of 0.05 mm/min. The “slice model” equation of Munz *et al*.^41^ was used to calculate K_Ic_.

## Additional Information

**How to cite this article**: Silvestroni, L. *et al*. Super-strong materials for temperatures exceeding 2000 °C. *Sci. Rep.* **7**, 40730; doi: 10.1038/srep40730 (2017).

**Publisher's note:** Springer Nature remains neutral with regard to jurisdictional claims in published maps and institutional affiliations.

## Supplementary Material

Supplementary Information
