# Supplementary material for: Super-strong materials for temperatures exceeding 2000 °C
Source: Sci Rep. 2017 Jan 19;7:40730. doi: 10.1038/srep40730 (PMC5244373; doi:10.1038/srep40730)
Supplement: Supplementary Information [file srep40730-s1.pdf]

Super-strong materials for temperatures exceeding 2000°C

Laura Silvestroni[11], Hans-Joachim Kleebe[2], William G. Fahrenholtz[3], Jeremy Watts[3]

[1]*CNR-ISTEC, Institute of Science and Technology for Ceramics, Via Granarolo 64, I-48018 Faenza, Italy*

[2]*TUD-IAG, Institute of Applied Geosciences, Schnittspahnstraße 9, D-64287 Darmstadt, Germany*

[3]*Department of Materials Science and Engineering, Missouri University of Science and Technology, Rolla, MO 65409*

SUPPLEMENTARY INFORMATION

THERMODYNAMICS
Zr-W-O system

According to some recent studies carried out on a ZrB_2 ceramic sintered with WSi_2,[18] it has been ascertained by TEM analysis that the oxygen partial pressure (PO_2) has a fundamental role in the nature of condensed phases that we find in the microstructure. To demonstrate that, we show in Fig. S1 the phase stability diagrams calculated by means of the commercial package HSC Chemistry v. 6.12 (Outokumpu research Oy, Pori, Finland). These maps are rather useful to define stability areas of condensed phases vs temperature and/or chemical potential for selected systems. Fig. S1 shows that at 1500°C W is stable for oxygen partial pressure below ~10^{-9}atm, whilst above this value liquid WO_3 is more favorable. This explains why W can survive in the form of nano-beads encased into the outermost ZrO_2 grains.

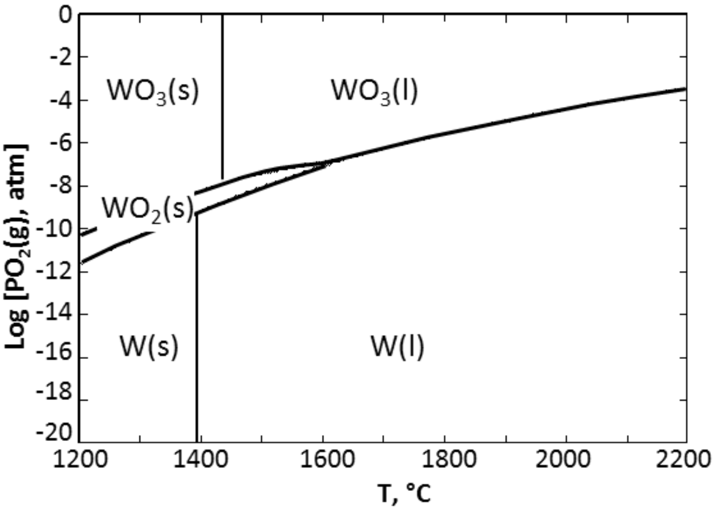

Fig. S1: Phase stability diagram of the W-O system as a function of oxygen partial pressure, PO_2(g), and temperature (T).

[1]Corresponding author: Laura Silvestroni
telephone: +39 546 699723
fax: +39 546 46381
e-mail: laura.silvestroni@istec.cnr.it
